# Synchronous invasive ductal carcinoma of the breast and clear cell renal carcinoma with rare presentation and behaviour: a case report with a literature review

**DOI:** 10.3332/ecancer.2020.1120

**Published:** 2020-10-08

**Authors:** Mona Ali Hassan, Najla Fakhrudiin, Fadi Farhat

**Affiliations:** 1 Department of Internal Medicine, American University of Beirut Medical Center, Beirut, Lebanon; 2 Department of Pathology, Hammoud Hospital University Medical Center, Sidon, Lebanon; 3 Department of Hematology and Oncology, Hammoud Hospital University Medical Center, Sidon, Lebanon

**Keywords:** synchronous tumours, clear cell renal cell carcinoma (RCC), syncytial-type giant cells, invasive ductal carcinoma

## Abstract

The presence of two or more primary tumours is a relatively uncommon phenomenon. Recently with the improvement of diagnostic modalities, such cases are increasingly reported in the literature. This paper presents a rare case of synchronous breast and renal tumour with unusual features including RCC metastasis to the duodenum and stomach, rapid recurrence of the tumour at the nephrectomy site, rapid renal cell carcinoma growth rate and the rare presence of syncytial-type giant cells in the renal cell tumour.

## Introduction

The incidence of multiple primary malignancies (MPM) which is generally defined as the occurrence of multiple malignancies that develop from different tissues with distinct morphologies has been currently increasing with a prevalence frequency of 2%–17%. This increase is correlated to multiple factors including old age, long-term side effects of chemotherapy and/or radiation therapy in cancer survivors, persisting effects of genetic and behavioural risk factors and improved diagnostic modalities [1]. MPM is classified by the North American Association of Central Cancer Registries into two categories:

1. Synchronous defined as two cancers occurring at the same time. The second tumour should be diagnosed within 2 months according to the Surveillance Epidemiology and End Results (SEER) programme.
2. Metachronous defined as cancer that follows in sequence the primary tumour and has 6 months’ duration or more, with the latter tumour being the more common one [2].

The International Association of Cancer Registries and International Agency for Research on Cancer (IACR/IARC) suggest the registration of synchronous tumours diagnosed in less than 6 months (or metachronous if more than 6 months) if arising in different sites.

Other difference between SEER and IACR is the fact that IACR considers tumours that occur in a different part of the same organ as a single tumour, whereas the SEER considers each one as a single tumour by itself; for example, in the colon, if we have primary tumours in different sites of the colon, SEER considers each one of them as single one, whereas IACR considers all of the tumours in the colon as one tumour [3].

Some cancers are more likely to occur together due to their common genetic backgrounds such as ovarian and breast cancers associated with BRCA mutation, or the multiple primary malignancies occurring in Von Hippel–Lindau or Lynch syndrome. Breast cancer is reported to occur synchronously with ovarian, colon, endometrial and vulvar cancer, whereas renal carcinoma occurs synchronously with other tumours but rarely with breast carcinoma [4]. In this paper, we report a rare case of synchronous invasive ductal breast carcinoma and renal cell carcinoma in a 64-year-old female, who had an unusual progression, behaviour and morphology of her disease.

## Case presentation

A 64-year-old female previously healthy, non-smoker, non-alcoholic, without a history of malignancy in her family presented to the practice in October 2016 with a left breast mass, and she was diagnosed with left breast invasive ductal carcinoma (IDC) positive for oestrogen and progesterone receptor and has no overexpression of HER-2. Tumour was locally advanced with clinically palpable axillary lymphadenopathy. The patient underwent a metastatic workup including CT brain, chest, abdomen and pelvis with whole-body PET/CT scans that showed a right kidney mass. An ultrasound-guided biopsy of the renal mass revealed a clear cell renal cell carcinoma confirmed by immunohistochemistry staining positive for CD10 and vimentin and negative for breast cell marker (GATA3), oestrogen and progesterone receptors. The renal cell carcinoma was considered as a second primary or as a concomitant tumour.

**Figure 1.**
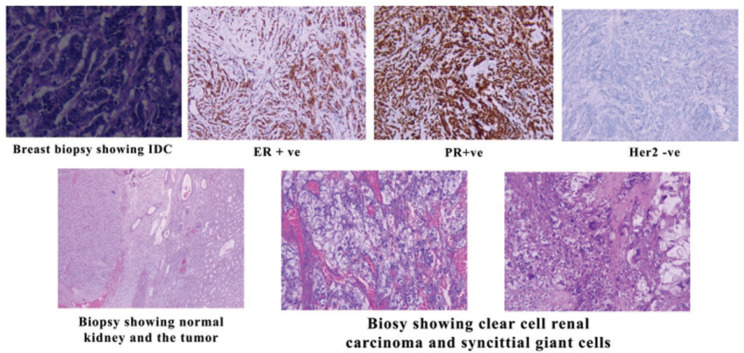
Biopsies of the IDC and RCC.

The patient only underwent right nephrectomy for the RCC, and she did not receive any further treatment. The tumour morphology demonstrated a predominance of low-grade clear cell renal carcinoma; however, the presence of scattered areas with syncytial-type giant cells raised the grade to 3/3 according to the WHO grading system (Figure 1). PET-scan showed uptake in the breast and the axillary area only and no metastasis; therefore, the pathological stage [tumour, nodes, and metastases (TNM)/American Joint Committee on Cancer (AJCC)] of the renal tumour was (pT3a N0 M0).

For her invasive ductal carcinoma, the patient received neoadjuvant chemotherapy with four cycles of TA (Taxotere and doxorubicin) followed by a left modified radical mastectomy and axillary lymph node dissection on May 2017. The breast tumour pathologic stage was (pT 1 (20 mm) N 2(5/16 +LNs) M0). Then, she received AC (adriamycin and cyclophosphamide) and continued adjuvant chemotherapy with weekly Taxol for 12 weeks, followed by radiotherapy and endocrine therapy.

**Figure 2.**
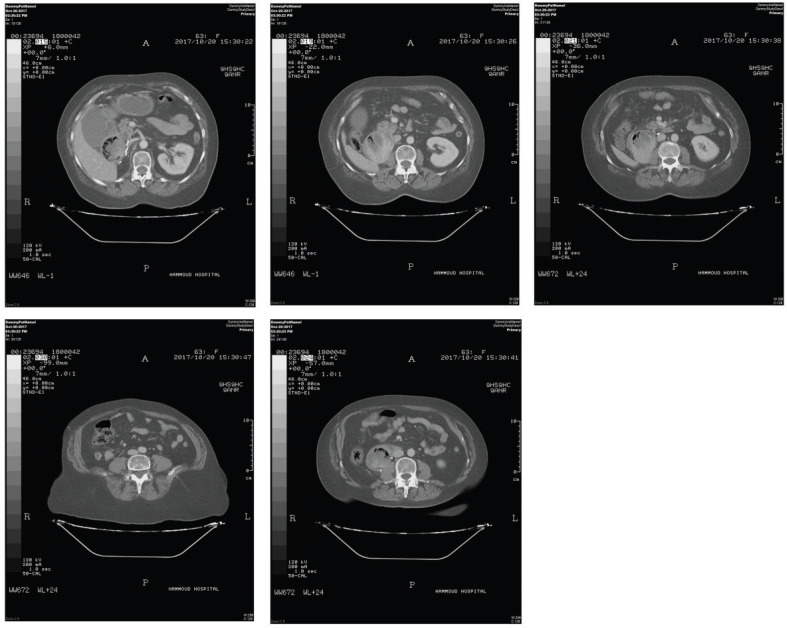
CT scan showing the duodenal mass separated from the recurrence in the nephrectomy fossa.

After the third Taxol chemotherapy cycle, she complained of recurrent vomiting and obstructive jaundice. Therefore, a CT scan of the abdomen and pelvis was done and showed stenosis at the duodenal orifice by a large duodenal mass causing significant distension of the second and third duodenal loop and a large mass at the right nephrectomy fossa. The duodenal mass was separated from the recurrence in the nephrectomy fossa as shown in Figure 2, and accordingly, metastasis was suspected, and the patient underwent gastrojejunostomy with choledojejunostomy and cholecystectomy to relieve the outlet obstruction. Because of the excessive tumour vascularity and tissue adherence, the tumour was non-resectable, and a biopsy was performed instead.

**Figure 3.**
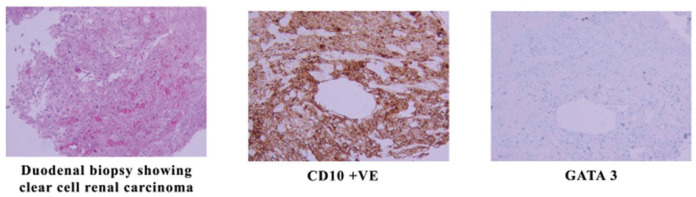
Biopsy from the duodenal mass showing RCC.

The pathology confirmed metastatic renal cell carcinoma involving both the stomach and the duodenum as shown in Figure 3, where immune stains were positive for CD10 and negative for GATA3.

**Figure 4.**
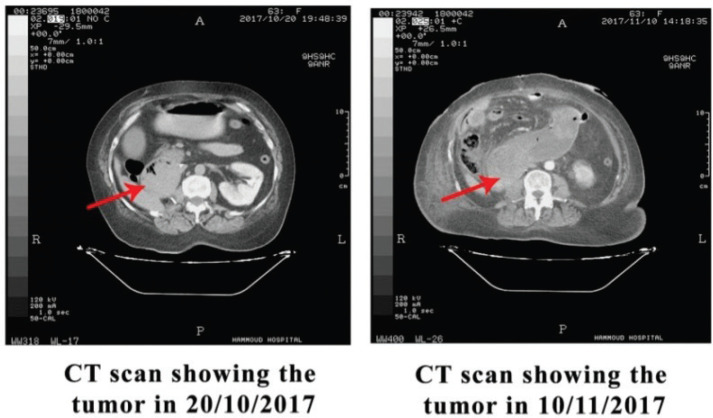
CT scan showing rapid tumour progression.

Postoperatively, the vomiting persisted along with a drop in haemoglobin and platelet level. Another CT scan of the abdomen and pelvis (Figure 4) was done 20 days postoperatively demonstrating a marked increase in tumour size with infiltration of D2–D4 duodenal bulb and extending to the proximal part of the jejunum (about 29 cm), thus a 13-cm increase in the tumour size, along with areas of hypodensities representing possible areas of haemorrhage that might explain her drop in haemoglobin, and no apparent bleeding haemolysis was evident. Unfortunately, the patient’s critically ill condition did not allow her to receive any kind of therapy for her RCC, and she died from an infection in the ICU shortly after the surgery.

## Discussion

The aetiology of multiple primary malignant tumours is complex, and it includes environmental factors such as tobacco, occupation, pollution and ultraviolet light, in addition to genetic predisposition, therapeutics (radiotherapy or chemotherapy), gender-specific factors and hormonal factors [5]. Synchronous breast cancer and RCC are very rare. Jiao *et al* [6] found, in a population-based study, eight cases with a prevalence of 13.1% of synchronous breast primaries with RCC. Most of those cases were limited to non-metastatic RCC and hormone receptor-positive IDC. Both tumours were treated with complete resection followed by chemoradiation and hormone therapy similar to the case, thus raising the possibility of a genetic link [2].

This dual malignancy case belongs to the synchronous category because both tumours are malignant, pathologically distinct from each other, and the possibility of metastasis was ruled out [7]. Moreover, this case has multiple unusual features that included RCC progression, behaviour and morphology.

The first unusual feature was the RCC metastasis to the duodenum and stomach. In general, 4% of RCC metastasises to the gastrointestinal tract. Metastasis to the duodenum is reported in 25 cases only with an incidence rate of 0.2% and 0.7% having gastrointestinal haemorrhage or obstruction (as reported in our case) as the most common clinical presentation [8]. Metastasis of RCC to the duodenum occurs via lymphatic and haematogenous spread usually or by direct infiltration; what may explain the duodenal involvement from the recurrent mass in the right nephrectomy bed in this case [9].

The second unusual feature was the fast recurrence of tumour at the nephrectomy site. The incidence of recurrence of RCC with no lymph node or distant organ metastasis at presentation is around 7% with a median time of 38 months for T1 tumours, 26% with a median time of 32 months for T2 disease and 39% with a median time of 17 months for T3 tumours (similar to this case) [10, 11]. The recurrence rate of RCC, in this case, is less than 12 months’ post-nephrectomy.

The third unusual feature was the fast growth rate. Clear cell renal carcinoma growth rate is 0.86 cm/year [11], and this case had a growth rate greater than 10 cm in 20 days, unexplained to the small areas of haemorrhage in tumour.

The fourth was the presence of syncytial-type giant cells, a rare presentation in RCC [12]. Cantalejo et al. have reviewed 55 RCC cases with different types of multinucleated giant cells (MGC). Only two of 55 of these RCC cases showed a syncytial type MGC. These cells were found to have the same immunophenotype as the original tumour cell, and thus, the author suggested a mononuclear tumour cell fusion process [13]. Syncytial-type giant cells are associated with aggressive behaviour as reported by Williamson *et al* [14].

## Conclusion

Currently, multiple primary malignancies are being encountered more frequently. Approaching these tumours may be complicated, but further studies including genetic and molecular profile analysis may help to understand their nature and clinical behaviour and provide more effective treatment modalities.

## Informed consent

Written informed consent was obtained from the patient who we report in this case.

## Conflict of interest

No conflict of interest is declared by the authors.

## Financial disclosure

There is no financial support.
